# A Short Comment on Michael Slote, “The Many Faces of Empathy”

**DOI:** 10.1007/s11406-016-9717-8

**Published:** 2016-04-30

**Authors:** R. Zaborowski

**Affiliations:** Department of Humanities, University of Warmia and Mazury, Olsztyn, Poland

**Keywords:** Michael Slote, Empathy, Feeling, Contagion, Feelings

## Abstract

ᅟThe comment discusses M. Slote's view on empathy as presented in his paper “The Many Faces of Empathy”. It is asked whether three forms of empathy he portrays are three separable concepts or three variants of the same concept of empathy.

In “The Many Faces of Empathy” Michael Slote treats two issues. He deals with empathy’s role in the theory of knowledge and with empathy’s role in speech acts. As it seems to me, he presents, all in all, three different faces of empathy, respectively in sections 1, 2, and 3–4.

In section 1 Slote understands empathy as “a direct way of knowing about other minds”. By doing so he says nothing about the affective element involved in empathy. This may mean that empathy is understood as cognitive rather than affective. Moreover, the kind of objects of knowledge grasped through empathy or the kind of acts resulting from empathy is not specified either. This makes room for a counterintuitive way of understanding empathy. For instance, an exceptionally sophisticated torturer or some other psychopath who efficiently control others because they perfectly read in their victims’ minds acquire a direct knowledge about other minds. Yet, neither the torturer not the psychopath is standardly considered as an empathic individual. They are both devoid of caring about others. While they are or can be extremely intelligent, they lack exactly the ability (necessary) to empathize even if they can coldly know about other minds.

When Slote says that empathy “helps us learn facts about the world”, he expands its meaning even more. Does it mean that these facts are not of special character, say, being directed at other persons’ feelings? (Slote says that “empathy helps to put us cognitively in touch with what others are feeling” but he does not develop this “feeling” element). This looks as if empathy is a way of knowing about other’s minds and other facts of the world independently of what results, if anything at all, from empathizing with them. Because empathy is a general way of learning about the world while several emotions are contributing to one’s knowledge too, but each of them in a specific way, would then be empathy a cognitive genus of what several emotions are just cognitive species?

Example Slote gives refers to osmosis and/or introjection or contagion rather than to empathy. He speaks about a child who learns fearing snakes because he empathically pick up on parents’ fear of snake. If empathy is indeed such an uncritical “way of learning about the world” (children have no “independent reason to doubt” as adults do, see below), its epistemic character is limited. Take a child who fears travelling in a plane because his parent does so. His learning can be misleading him in his life and if we call empathy the way he acquired his knowledge in this respect, its harming role is to be conjectured. This is what Slote hints at when he speaks about (paranoiac) identification with or appropriation of other people’s belief or attitude. There is not, however, a clear divide between such distinct phenomena as paranoia and psychopathy, on the one hand, and empathy, on the other. Traditionally empathy has been understood as morally beneficial and as exclusive with psychopathy. Therefore, “empathy [that] serve us badly” and, accordingly, empathy not “fully reliable” is a concept that unless misleading should be better spelled out. Otherwise how one can subscribe to the view that “empathy can (often) help us identify those who need our help more immediately or quickly than other modes of cognition, and this clearly makes empathy epistemically relevant to the moral life”? One of two things: either empathy is morally relevant and then cannot be the same as getting into the heads of other people unqualifiedly and for any purpose whatsoever, or it is “a direct way of knowing about other minds” regardless of my intention stemming from empathizing but then empathy is relevant to moral only contingently. If Slote insists on “the acquisition of empirical knowledge or justified beliefs about the world”, it should be clarified in what way acquired beliefs are justified - surely not always in the sense of being accurate or appropriate - and whether the acquisition is or is not essentially and inseparably related to undertaking an act or being motivated to act in a special way.

In section 2 Slote speaks about “being warmed by warmth or chilled by cold-hearted (or heartless)” as “an empathic process”. This is another face of empathy: “empathically registered warmth and chill as, respectively, states of moral approval and disapproval”. Empathy looks now as an autonomous and (always?) right awareness of what is going on in the world. Whereas in section 1 empathy was about fully accepting or adopting one’s attitude, uncritically and at risk of acquiring a false conviction, here, in section 2, empathy is about decoding the meaning of “someone’s actions or attitude by empathically taking in the warmth or coldness that person is displaying in their actions”.

In section 3 a third face of empathy amounts to “recogniz[ing] the feelings of others”. Slote applies the concept of empathy so understood to “what people say”, which, in turn, is manifest of “what they are thinking, wanting, or feeling”. I can agree with Slote that “empathy enters into the functioning of assertion and asking a question as speech acts”, however, it is useful to know if speech act as object of empathy is isolable from other, non-propositional or even non-verbal signs that accompany asserting or asking a question. For instance, if you are insincere or joking, I often know about it not from the content of assertion or question but from the tone of your voice, your countenance etc. And bodily expression can tell even more. I suppose the higher is the ability to empathize, the more accurate is recognition of what the speaker not only asserts or ask but also, or first of all, intends to assert or to ask. Example provided by Slote imply that he does not consider empathizing in this light, taking any assertion or question bona fide.

In his last section Slote comes back to empathizing with someone else’s fear. Yet, whereas in section 1 it was child’s empathizing with parent’s fear of snake, now it concerns an adult’s empathizing with child’s fear of worms. He says: “if one empathically picks up on a child’s fear of worms but has independent reason to doubt the dangerousness of worms, that may undercut the acquisition of any knowledge of or reasonable belief about what the world is like”. Again, this is not what I am used to consider as empathy which is not about, in the case mentioned, the dangerousness of the world as apprehended mistakenly by the child in question; it is rather about feelings of the child. His fear may be perceptible to many, but only he who empathizes with him not only knows about his fear but also understands it. And not only he understands it but also he is inclined to act in such a way as to improve that child’s condition if this is morally possible or necessary (there is no need of improving a condition of a happy person whom I empathize with or if I consider that the child will be better off if he learns how to face his fear of that particular kind). Despite of possessing “independent reason to doubt the dangerousness of worms”, I still understand why and how the child fears worms. Finally, if we agree that empathy prompts to caring, an empathic person is the one who is ready to help. One more time, the more empathic the person is, the better he will help the child.

But now we are given what was absent is section 1, this is an epistemic criticism going together with empathy. Why this difference? Criticism regarding the object of feeling whom I empathize with is irrelevant because child’s fear as such is a different object of empathizing than the object of this child’s fear, while in the case of the dangerousness of the world the child has no resources to be critical of parent’s fear. Are they two distinct concepts of empathy of which one is epistemically critical while the other uncritical or do we deal with a single concept of empathy which is or is not accompanied by an epistemic criticism? The two examples are so different that I think that they refer to two distinct notions of empathy, not to two faces of empathy, or, as it is presented by Slote, empathy plays a different role in children and in adults.

I do not know either how much such categories as osmosis, contagion, empathy, sympathy and transmission, all used by Slote, converge and diverge (please remark that in one sentence Slote uses empathy and sympathy interchangeably) and how each of them operate in the realm of epistemology and moral philosophy. More importantly, given three different descriptions of empathy I wonder if they are three faces of one empathy concept or three different views of empathy, in fact, three distinct concepts. If the former, it would be required to explain how they concur, given their contrasting or contradictory features.

